# Visual deprivation selectively reshapes the intrinsic functional architecture of the anterior insula subregions

**DOI:** 10.1038/srep45675

**Published:** 2017-03-30

**Authors:** Lihua Liu, Congcong Yuan, Hao Ding, Yongjie Xu, Miaomiao Long, YanJun Li, Yong Liu, Tianzi Jiang, Wen Qin, Wen Shen, Chunshui Yu

**Affiliations:** 1 Department of Radiology, Tianjin Key Laboratory of Functional Imaging, Tianjin Medical University General Hospital, Tianjin 300052, China; 2 Department of Radiology, Tianjin First Central Hospital, Tianjin 300192, China; 3 School of Medical Imaging, Tianjin Medical University, Tianjin 300070, China; 4 Brainnetome Center. Institute of Automation, Chinese Academy of Sciences, Beijing 100190, China; 5 National Laboratory of Pattern Recognition, Chinese Academy of Sciences, Beijing 100190, China; 6 CAS Center for Excellence in Brain, Science and Intelligence Technology, Institute of Automation, Chinese Academy of Sciences, Beijing 100190, China; 7 University of Chinese Academy of Sciences, Beijing, 100049, China; 8 Key Laboratory for NeuroInformation of the Ministry of Education, School of Life Science and Technology, University of Electronic Science and Technology of China, Chengdu 625014, China; 9 The Queensland Brain Institute, University of Queensland, Brisbane, QLD 4072, Australia

## Abstract

The anterior insula (AI) is the core hub of salience network that serves to identify the most relevant stimuli among vast sensory inputs and forward them to higher cognitive regions to guide behaviour. As blind subjects were usually reported with changed perceptive abilities for salient non-visual stimuli, we hypothesized that the resting-state functional network of the AI is selectively reorganized after visual deprivation. The resting-state functional connectivity (FC) of the bilateral dorsal and ventral AI was calculated for twenty congenitally blind (CB), 27 early blind (EB), 44 late blind (LB) individuals and 50 sighted controls (SCs). The FCs of the dorsal AI were strengthened with the dorsal visual stream, while weakened with the ventral visual stream in the blind than the SCs; in contrast, the FCs of the ventral AI of the blind was strengthened with the ventral visual stream. Furthermore, these strengthened FCs of both the dorsal and ventral AI were partially negatively associated with the onset age of blindness. Our result indicates two parallel pathways that selectively transfer non-visual salient information between the deprived “visual” cortex and salience network in blind subjects.

The salience network (SN)^1^ has recently attracted strong interest in many neuroimaging studies for both normal subjects and patients with neuropsychiatric^2^,^3^, neurodegenerative^4^,^5^,^6^, and neurodevelopmental disorders^7^. The anterior insula (AI) is one of the core hubs of the SN that serves to identify the most relevant salient stimuli and forward them to higher cognitive regions to guide behaviour^8^. Recent studies have proposed that it can be further segmented into two functional segregated sub-regions: the dorsal AI, which serves to process cognitive related information, and the ventral AI, which preferentially processes social-emotional information^9^,^10^,^11^,^12^. As the AI can receive different types of sensory stimuli and integrate them for cognitive awareness^13^,^14^, it would be interesting to understand the effect of different types of sensory experience on the development and plasticity of AI functional organization.

Sensory deprivation, such as blindness, is a good model to explore the neuroplastic capabilities of the brain^15^,^16^,^17^. Perception is the foundation of cognition, emotion and action. In contrast to sighted controls, blind individuals must rely more heavily on hearing and touch to capture salient cues from non-visual environment, which induces compensatory changes in the abilities of non-visual perception. In fact, abundant studies have demonstrated that blind people have altered auditory^18^,^19^,^20^, olfactory^21^,^22^ and tactile perceptual abilities^23^,^24^,^25^, as well as improved non-visual attentional^26^,^27^ and work memory performance^26^,^28^, etc. In addition to the non-visual behavioural changes, cross-modal involvement of the visual areas in processing non-visual stimuli were also identified by early studies, which included tactile^29^,^30^,^31^,^32^,^33^, auditory^29^,^34^,^35^,^36^,^37^, and olfactory^38^ perception, and even higher cognitive processing driven by these non-visual stimuli such as language^39^,^40^, attention^26^,^27^ and working memory^26^,^28^. All these findings support that the “visual” cortical organization of blind individuals is adaptively reshaped to process non-visual task-related stimuli to perceive the external environment.

Because the AI is one of the core hubs that serve to identify the salient stimuli from different sensory modalities and integrate them for cognitive awareness^13^,^14^, changes in sensory inputs (both modality and strength) would theoretically reshape it to adapt to the environment. Several studies have indicated the plastic potential of the AI in response to sensory deprivation. For example, early deaf individuals demonstrate stronger activation of the AI during visual short-term verbal memory task^41^,^42^, as well as a strengthened functional connectivity (FC) between the AI and the superior temporal sulcus, where cross-modal processing of the visual spatial working memory have been identified compared with hearing subjects^43^. Compared with the sighted subjects, the congenitally blind (CB) individuals demonstrate decreased regional homogeneities of the AI^44^. All these findings support that SN may play important roles in the neural reorganization following sensory deprivation. In a recent study using independent component analysis (ICA), Wang *et al*. reported increased spontaneous functional connectivity within the salience network (SN), as well as enhanced internetwork connectivity between the SN and the frontoparietal network (FPN)^45^ in the CB. However, that study only focused on the large-scale reorganisation of spontaneous functional coupling between the SN and other networks, while did not clarify which of the SN hubs (for example, the AI) is specifically reshaped in adapt to the visual deprivation; moreover, that study only explored the alterations in CB individuals, thus it is still unknown if individuals with different onset age of blindness shows similar (or diverse) alterations in spontaneous functional organization of AI-related network.

Early studies have shown that the onset age of blindness has a significant influence on the structure and function of the visual pathways, including cortical thickness^46^,^47^,^48^, grey matter volume^44^, cross-modal activity pattern^15^,^49^, baseline regional activity and metabolism^44^,^50^,^51^, and FC and functional connectivity density^45^,^52^,^53^,^54^, etc. These studies indicate that the structural and functional reorganization of the brain after visual deprivation is the complex contribution of development, experience-dependent plasticity and degeneration factors^55^. And onset of age of blindness within or after the limited sensitive period may alter the balance between these three mechanisms. Specifically, the CB or early blind (EB) individuals who lost their sight within the sensitive period may be modulated by developmental, experience-dependent plastic, and degenerative mechanisms, while the late blind (LB) individuals who lost their sight after the sensitive period is mainly modulated by experience-dependent plastic and degenerative mechanisms^15^. Thus, the second question needs to be clarified in this study is whether developmental factor contributes to the spontaneous functional reorganization of AI-related network in the blindness.

Finally, as proposed by early studies^9^,^10^,^11^,^12^, the dorsal and ventral AI selectively prefer to process cognitive and social-emotional stimuli, respectively, in the sighted subjects. In accordance, blind subjects exhibit changed perceptual performance, with both non-emotional^18^,^19^,^20^ and emotional^56^ conditions. Moreover, in both conditions, the visual areas were cross-modally involved^15^,^16^,^56^, and the blind subjects showed stronger BOLD responses than did the sighted subjects in the right amygdala in fearful/angry versus neutral conditions^56^. Thus, the third interesting question is that whether the functional networks of the dorsal and ventral AI are differentially reshaped for their specification in processing the non-motional and motional salient stimuli.

In the present study, we recruited a relatively large number of blind subjects to investigate the effect of long-term visual deprivation on the reorganization of the intrinsic functional network of AI subregions. We first hypothesized that the resting-state FCs of the AI with the visual pathway were strengthened based on early studies showing changed non-visual perceptual capabilities^19^,^20^,^23^,^24^,^25^,^57^ and cross-modal plasticity of the visual areas^29^,^31^,^58^,^59^,^60^ in the blind subjects, and increased FC between the AI and auditory cortex in the early deaf subjects^43^. Second, because the dorsal and ventral AI serve to different types of salient stimuli, we also hypothesized that the FC of these two sub-regions may be differentially reshaped. Finally, we hypothesized that developmental factor may contribute to the changes in FC of AI sub-regions in the blindness.

## Results

### Interactions between the groups, AI locations and hemispheres

**Figure 1:**
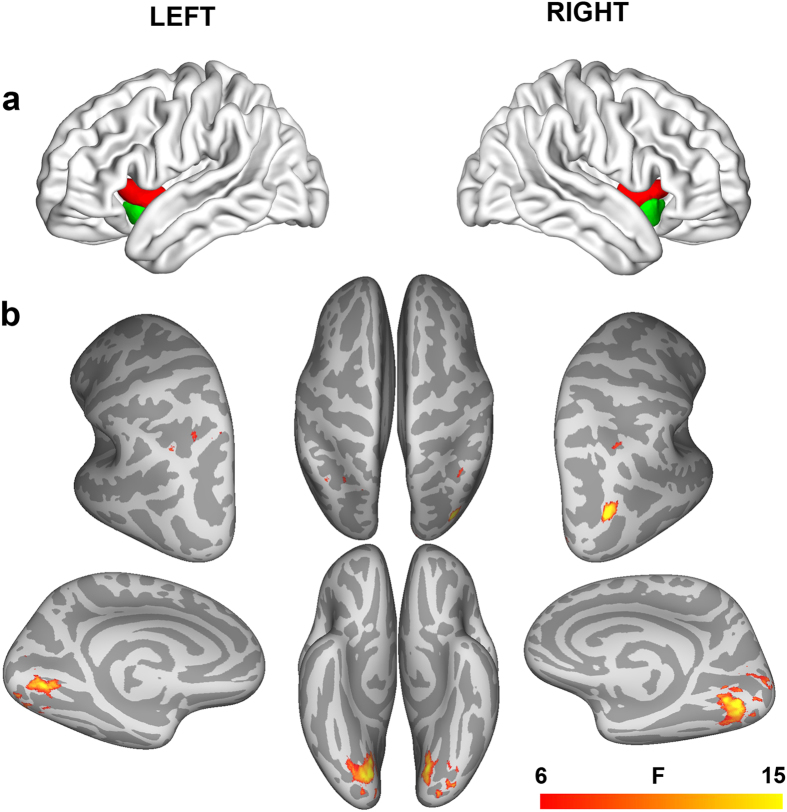
Interactions between groups and locations of anterior insula on the resting-state functional connectivity. A mixed-model ANOVA was performed with the groups (CB, EB, LB and SC) as between-subjects effect, and the hemispheres (left versus right AI) and locations (dorsal versus ventral AI) as within-subjects effects (q < 0.01, FDR corrected). Color bar represents the F value. (**a**) The AI subregions used for calculation of the FC were obtained from an early study by Deen and Pelphrey *et al*.^11^ (**b**) ANOVA revealed significant interactions between groups and AI locations, which were located in the bilateral IPA, CalS and LG, and MOG. Abbreviations: AI = anterior insula, ANOVA = analysis of variance, CalS = calcarine sulcus, CB = congenitally blind, EB = early blind, FC = functional connectivity, FDR = false discovery rate, IPA = intraparietal area, LB = late blind, LG = lingual gyrus, MOG = middle occipital gyrus, SC = sighted controls.

As shown in Fig. 1, a 4 groups (CB, EB, LB, and SC) x 2 hemispheres (left versus right side) x 2 AI locations (dorsal versus ventral area) mixed-model analysis of variance (ANOVA) revealed significant interactions between the groups (CB, EB, LB and SC) and AI locations (dorsal versus ventral area) with respect to the FC (q < 0.01, false discovery rate [FDR] corrected), indicating diverse inter-group differential patterns in the resting-state FC between the dorsal and ventral AI. Brain regions demonstrating significant interactions were mainly located in the bilateral intraparietal area (IPA), calcarine sulcus (CalS) and lingual gyrus (LG), and right middle occipital gyrus (MOG) (Table 1). We did not identify any significant interaction between the groups and hemispheres (left versus right AI), or among the groups, hemispheres and locations.

### Inter-group comparison on the FC of the dorsal AI

**Figure 2:**
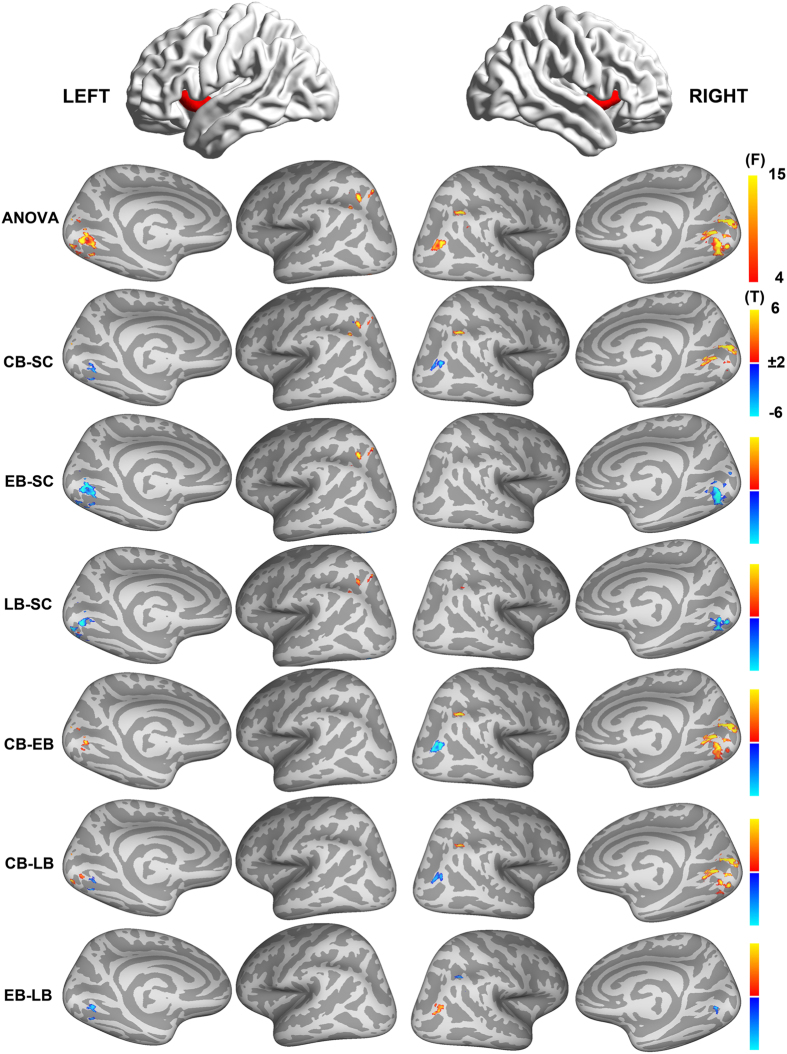
Intergroup differences in functional connectivity of the dorsal anterior insula. A one way ANOVA was performed to test inter-group differences in FC of the dorsal AI within the brain regions that showed significant group × AI location interactions (q < 0.01, FDR corrected). The first row represents the AI subregions. The second row represents the findings of one-way ANOVA, and the color bar in this row represents F value. The remaining rows represents the paired-wise comparisons between the 4 groups, and the color bar represents T value. Abbreviations: AI = anterior insula, ANOVA = analysis of variance, CB = congenitally blind, EB = early blind, FC = functional connectivity, LB = late blind, SC = sighted controls, FDR = false discovery rate.

Additional one-way ANOVA and post-hoc inter-group comparisons of the FC of the dorsal AI are shown in Fig. 2(q < 0.01, FDR corrected). For the dorsal AI, there were significant inter-group differences in FC between the dorsal AI and bilateral IPA, CalS and LG, and right MOG (Supplementary Figure S1). Post hoc analyses revealed that the CB had a higher dorsal AI FC with the bilateral IPA and CalS, while a lower FC with the left LG and right MOG than the SC; the EB and LB had higher dorsal AI FC with IPA, while lower FC with bilateral LG than the SC. In comparisons within the blindness group, the CB had higher dorsal AI FC with the bilateral CalS and right IPA, while lower FC with left LG and right MOG than the EB and LB; the EB showed a higher dorsal AI FC with the right MOG, and a lower FC with the bilateral LG than the LB.

### Inter-group comparison of the FC of the ventral AI

**Figure 3:**
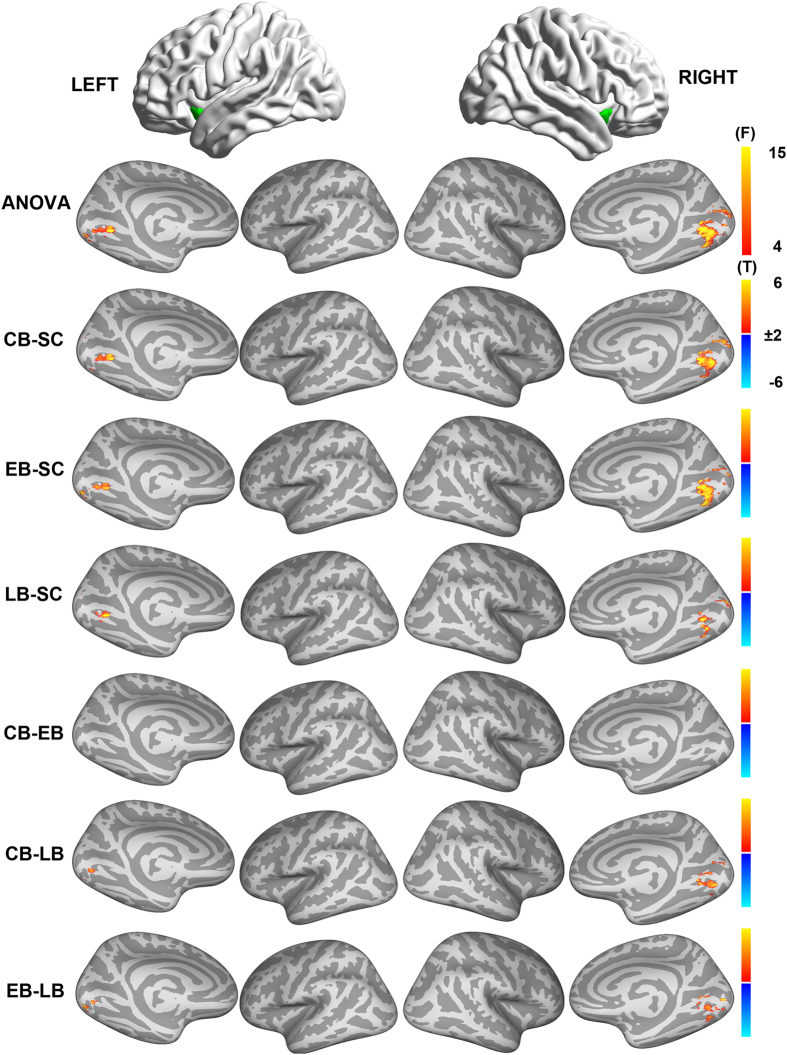
Intergroup differences in functional connectivity of the ventral AI. A one way ANOVA was performed to test inter-group differences in FC of the ventral AI within the brain regions that showed significant group × AI location interactions (q < 0.01, FDR corrected). The first row represents the AI subregions. The second row row represents the findings of one-way ANOVA, and the color bar in this row represents F value. The remaining rows represents the paired-wise comparisons between the 4 groups, and the color bar represents T value. Abbreviations: AI = anterior insula, ANOVA = analysis of variance, CB = congenitally blind, EB = early blind, FC = functional connectivity, LB = late blind, SC = sighted controls, FDR = false discovery rate.

Additional one-way ANOVA and post-hoc inter-group comparison of the FC of the ventral AI are shown in Fig. 3 (q < 0.01, FDR corrected). For the ventral AI, there were significant inter-group differences in FC at the bilateral CalS and LG. Post hoc analyses further revealed that all the blind groups had a higher ventral AI FC with the bilateral LG and CalS compared with the SC. In comparisons within the blindness group, both the CB and EB showed higher FC with the right CalS and bilateral LG than the LB, while there were no significant differences in the FC between the CB and EB.

### Correlations between the FC of AI subregions and onset age of blindness

**Figure 4:**
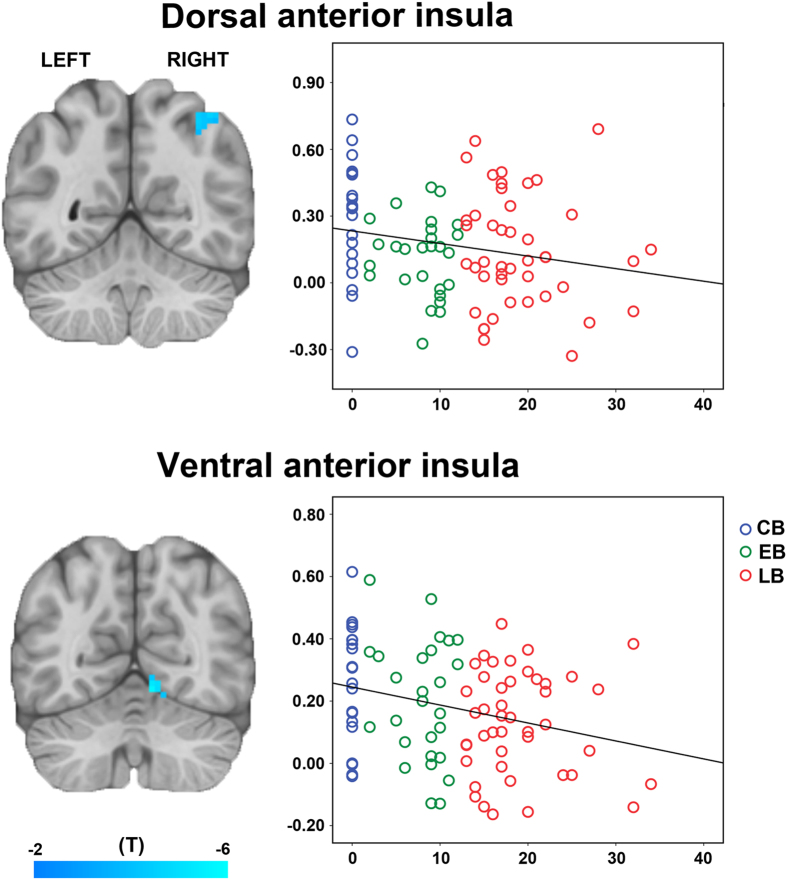
Relationship between functional connectivity of anterior insula subregions and the onset age of blindness. voxel-wise regression analyses demonstrated significant negative correlations between the onset age of blindness and FC of right IPA with the dorsal AI, and between the onset age of blindness and FC of right LG with the ventral AI (q < 0.01, FDR corrected). Color bar represents the T value. The scatter plots (right panels) represent the association between the onset age of blindness and mean FC of the visual ROIs that showing significant correlations with the onset age. Abbreviations: AI = anterior insula, FC = functional connectivity, FDR = false discovery rate, IPA = intraparietal area, LG = lingual gyrus.

Finally, voxel-wise regression analyses revealed significant negative associations in FC between the dorsal AI and right IPA, and between the ventral AI and right LG (q < 0.01, FDR corrected) with the onset age of blindness (Fig. 4).

## Discussion

In this study, we aimed to clarify the effect of long-term visual deprivation on the reorganization of the intrinsic functional network of AI subregions, which are the core hubs of the salience network. We found that the resting-state functional network of the dorsal and ventral AI subregions were selectively reshaped after blindness in all of the blind groups: The functional connectivities of the dorsal AI were strengthened with the dorsal visual stream in the blind, while weakened with the ventral visual stream; in contrast, the functional connectivity of the ventral AI was strengthened with the ventral visual stream in the blind. Furthermore, the onset of age of blindness had significant impact on the intrinsic functional architecture of both the dorsal and ventral AI. Our findings outlined two parallel strengthened pathways to AI subregions after visual deprivation, which may contribute to transmitting non-visual salient information between the deprived “visual” cortex and the SN.

One of the important findings was the strengthened functional connectivity of the AI subregions with visual areas in blind subjects irrespective of the onset of age of blindness. Early studies have indicated that the “deprived” visual areas indeed participate in the cross-modal processing of tactile/auditory stimuli^29^,^30^,^31^,^32^,^33^,^34^,^35^,^36^,^37^. Furthermore, the direct or indirect neural pathways conveying non-visual information from the subcortical nuclei or primary auditory/sensory cortex to the visual areas have been frequently reported to be rewired or strengthened after blindness (please see the review by Qin *et al*. for detail)^61^. However, it is still unknown whether the pathway transferring the processed signals from the “deprived” visual area to the high-level cognitive cortex is also reshaped by long-term visual deprivation. The present study provided additional evidence supporting strengthened functional links between the visual stream and the salient core hubs, which may partly interpret the compensatory changes in non-visual perceptual^18^,^19^,^20^,^23^,^24^,^25^ and cognitive performance in blind individuals^26^,^27^,^28^. This inference was supported by a recent serial study by Ding *et al*., who reported cross-modal activation of auditory regions during visuospatial working memory in early deafness^42^; moreover, strengthened functional connectivity has been shown between these reshaped auditory regions and the AI in early deafness, and the strengthened functional connectivity is significantly associated with the working memory performance^43^. Because the present study did not acquire the behaviour information, the direct association between the visual-AI functional coupling and the non-visual functions requires future clarification.

Notably, we did not find a significant change in functional coupling between the auditory/tactile cortex and AI in the blind. In combination with previous studies showing strengthened casual flow from the auditory cortex to the visual cortex^15^,^62^, visual deprivation might reshape the indirect pathway (for example, auditory-visual-AI) rather than the direct pathway (for example, auditory-AI) that transmit the non-visual information to the salience network. These results further support the functional significance of the visual areas in processing and conveying non-visual signals^29^,^34^,^35^,^36^,^61^.

The present study further highlighted the following differentially changed patterns in the functional connectivity of the dorsal and ventral AI: strengthened FC of the dorsal AI with dorsal visual stream and weakened FC with the ventral visual stream, while strengthened FC of the ventral AI with the ventral visual stream. Previous neuroimaging studies have proposed that the dorsal and ventral AI preferentially process cognitive and social-emotional stimuli, respectively, in the sighted subjects^9^,^10^,^11^,^12^. Anatomical and functional evidence also support the functional preferences for the dorsal and ventral visual stream, respectively. The dorsal visual stream (also known as the “where” stream) travels along the dorsal visual areas to the dorsal prefrontal cortex. It preferentially processes visual spatial-related perception and higher-level cognition^63^,^64^,^65^. The ventral visual stream (also known as the “what” stream) travels along the ventral visual areas and inferior temporal regions to the ventral prefrontal cortex and prefers to process category-related visual information^63^,^66^. The ventral visual stream also has strong connections with the medial temporal lobe and limbic system (such as the amygdala), and plays important roles in social-emotional processing^67^,^68^,^69^. After visual deprivation, blind subjects demonstrate compensatory changes in non-visual perceptual performance in different dimensions, including spatial^20^,^27^,^36^, category 24,57,70,71, and emotion^56^, etc. Furthermore, early studies had revealed that the visual areas of the blindness preserved their abstract functional specialization for processing non-visual information, such as dorsal visual areas for spatial perception^15^,^36^,^72^ and the ventral visual areas for object perception^60^,^73^. Thus, the differentially strengthened patterns of functional coupling of the dorsal and ventral AI found in the present study may outlined two parallel strengthened pathways that transfer non-visual salient information between the deprived “visual” cortex and the salience network in the blind subjects: a dorsal pathway conveying non-visual spatial information between the dorsal visual stream and the dorsal AI, and a ventral pathway transferring non-visual object and social-emotional information between the ventral visual stream and the ventral AI. Because functional connectivity cannot provide directional information, the present study cannot infer the top-down or bottom-up relationships between the visual cortex and AI, which should be clarified using non-invasive (such as the dynamic causal model) or invasive (such as transcranial magnetic stimulation or lesion analyses) causal analyses techniques. In addition to the strengthened functional coupling between the dorsal AI and dorsal visual stream, we also found decreased functional coupling between the dorsal AI and the ventral visual stream. Notably, as shown in Supplementary Figure S1, the MOG that showed decreased FC with the dorsal AI in the CB compared with the SC were partial overlapped with the visual area LOC (lateral occipital complex) according the PALS-12 atlas. Area LOC preferentially processes object information^74^,^75^ and is generally categorized as the ventral stream. Besides, the majority of the MOG is outside the traditional retinotopic areas and is adjacent to several dorsal visual areas, thus this ROI may also play roles in communicating the ventral and dorsal stream. The dorsal and visual streams have direct or indirect (for example, the MOG in present study) reciprocal connections with each other^76^,^77^, which are critically important to integrate the spatial and object information to form complete knowledge of the external world^64^,^78^,^79^. The inverse changes in the FC of the dorsal and ventral streams with the dorsal AI might indicate a deficiency of blind subjects in integrating the spatial and object information for cognitive processing, which should be clarified in later studies.

Another important point of our findings was that the FC change patterns of both the dorsal and ventral AI were dramatically similar among the CB, EB and LB, although the effect of onset of age of blindness cannot be ignored. The functional and structural reorganization of visual areas and other higher cortices after blindness are driven by complex influences from developmental, experience-dependent plasticity and degenerative factors^55^,^80^,^81^. Early studies have demonstrated that onset age of blindness have significant impacts on the functional and structural architecture of blind brain, indicating developmental factors play important roles in reshaping visual areas^15^,^82^,^83^. It can explain higher functional connectivity between the AI subregions and the visual streams in blind individuals who lost vision at their earlier age (i.e., the CB or EB) than those who lost vision at elder age (i.e., the LB). However, it cannot explain the most consistent findings of strengthened functional synchronization of AI in all of the blind subjects, because for the LB, the brain architecture is generally considered matured before visual loss. In fact, the visual areas have been acknowledged to be supramodal in nature, which means that the functional specializations of the visual areas are task-dependent rather than sensory-dependent, and visual experience is not necessary to develop and mould the functional architecture of visual areas because of existing connections from other sensory modalities. Thus, the common findings highly suggest that the reorganization of the intrinsic functional architecture of AI of blind subjects was mainly driven by combined influences by experience-dependent and degenerative factors rather than developmental factors: blind subjects would rely more heavily on the remnant auditory/tactile inputs and perform more exercise than would sighted subjects, and this long-term training effect strengthens the existing links between the deprived “visual” areas and the AI.

## Conclusions

In summary, we found that the intrinsic functional networks of AI subregions were selectively reshaped after blindness, exhibiting a strengthened FC of the dorsal AI with the dorsal visual stream, and a strengthened FC of the ventral AI with the ventral visual stream. These findings suggest two parallel dorsal and ventral pathways that selectively transfer non-visual salient information between the deprived “visual” cortex and salience network in the blind subjects. Moreover, the common findings in bind subjects irrespective of the age at vision loss indicates that the reorganization of the intrinsic functional architecture of AI of blind subjects was mainly driven by combined influences by experience-dependent and degenerative factors, and that the onset of age of blindness also exerts minor effects on this process.

## Methods

### Participants

In total, 141 right-handed subjects comprising 20 CB (12 males, blind since birth, age range from 20 to 39 years old, mean age 26.6 ± 5.0 years old), 27 EB (20 males, onset age equal to or lower than 12 years old, age range from 20 to 45 years old, mean age 28.9 ± 7.4 years old), 44 LB (30 males, onset age higher than 12 years old, age range from 20 to 45 years old, mean age 30.9 ± 6.5 years old) and 50 SC subjects (33 males, age range from 20 to 45 years old, mean age 28.8 ± 7.00 years old) were involved in further analysis. There were no significant differences in either gender (chi-square test, χ2 = 0.63, P = 0.889) or age (one-way analysis of variance, F = 2.07, P = 0.107) among the 4 groups. Notably, the same cohort was reported in a previous study. For a detail description of the demographic information of the participants please see the study by Jiang *et al*.^44^ and Supplementary Table S1. This study was approved by the Medical Research Ethics Committee of Tianjin Medical University General Hospital. The experiments were carried out in accordance with the relevant guidelines and regulations of the Ethics Committee. Written informed consent was obtained from all the participants after a complete description of the study prior to the experiment.

### Image data acquisition

MRI data were obtained using a 3.0-Tesla MR scanner (Trio Tim system; Siemens, Erlangen, Germany) with a 12-channel head coil. Structural images were acquired using 3D magnetization-prepared rapid-acquisition gradient echo (MP-RAGE) sequences with the following parameters: repetition time (TR)/echo time (TE)/inversion time = 2000/2.6/900 ms, flip angle = 9 degree, matrix = 256 × 224, field of view (FOV) = 256 mm × 224 mm, 176 continuous sagittal slices with a 1-mm thickness, resulting in a voxel size of 1 × 1 × 1 mm^3^. The resting-state fMRI data were acquired with a gradient-echo echo-planar imaging (GRE-EPI) sequence. The acquisition parameters included the following: TR/TE = 2000/30 ms, flip angle = 90 degree, matrix = 64 × 64, FOV = 220 mm × 220 mm, 32 axial slices with a 3-mm slice thickness and a 1-mm gap, resulting in a voxel size of 3.4 × 3.4 × 3 mm^3^. During the fMRI scans, all subjects were instructed to remain their eyes closed, to keep motionless, to think of nothing in particular, and to not fall asleep.

### Whole brain functional connectivity analysis

#### Preprocessing of resting-state fMRI data

The resting-state fMRI data were preprocessed using Statistical Parametric Mapping (SPM8, http://wwwfil.ion.ucl.ac.uk/spm) with the following steps: The first 10 volumes were discarded to stabilize the MR signal and to allow the participant to adapt to the scanning noise. The remaining 170 volumes were slice-timing corrected. Subsequently, rigid realignment was performed to estimate and correct for the motion displacement. The frame-wise displacement (FD) was also calculated based on the head motion parameters^84^. The data of each individual were within the acceptable head motion thresholds with the maximum displacement in any direction was <2 mm, the maximum rotation <2.0°, and average FD <0.5. Three LB and 1 SC subjects were excluded because the durations of unspiked fMRI timecourse of them were lower than 5 minutes. One-way ANOVA showed no statistical inter-group differences in either mean FD (F = 0.99, P = 0.40) or percentage of spike volumes (F = 1.70, P = 0.17) in the remaining subjects. Next, several nuisance covariates were regressed out from the motion corrected fMRI data, including the mean signals of the white matter and cerebrospinal fluid, six rigid motion parameters and their first-level time derivatives, and the spike volumes with FD >0.5 to further diminish the possible influence of head motion. We used a two-step coregistration method to transform the regressed fMRI data into the MNI space. First, the mean realigned fMRI images were affinely coregistered with the individual structural images; then the structural images were affinely coregistered with the standard MNI T1-weighted template. The generated parameters for these two coregistration steps were concatenated and used to normalize the regressed fMRI data. The normalized fMRI data were resampled into a voxel size of 3 mm × 3 mm × 3 mm. Finally, the normalized fMRI volumes were smoothed with a Gaussian kernel of 6 mm × 6 mm × 6 mm full-width at half maximum (FWHM).

#### Functional connectivity calculation

The seeds for bilateral dorsal and ventral AI were obtained directly from a recent work by Deen and Pelphrey *et al*.^11^, who parceled the insula into 3 sub-regions based on the functional connectivity patterns. Then, the mean timecourses of each seed were extracted, and the Pearson correlation coefficients between the timecourses of the seeds and those of each voxel of the whole brain were computed and converted to z values using Fisher’s r-to-z transformation to improve the normality.

### Statistical analysis

In this study, we mainly focused on the effect of onset age of blindness and AI subregions on the resting-state functional connectivity. Thus, a 4 groups (CB, EB, LB, and SC) × 2 AI hemispheres (left versus right AI) × 2 AI locations (dorsal versus ventral AI) mixed-model ANOVA was performed based on a general linear model (GLM) within a grey matter mask. We considered the groups (CB, EB, LB and SC) as the between-subjects effect, and the hemispheres (left versus right AI) and locations (dorsal versus ventral AI) as the within-subjects effects. Notably, in the present study, we were only interested in the interaction effects between group and location, between group and side, and among the three factors. Multiple comparisons were corrected using voxel-wise false discovery rate (FDR) methods (q < 0.01). If any significance existed in each of these interactions, voxel-wise post hoc analyses were further performed to clarify the potential differences in FC between each pair of the 4 groups at each level of other factors within the searching voxels that showed statistically significant interaction effects (q < 0.01, FDR correction). Finally, voxel-wise regression analyses were carried out to investigate the association between the onset age of blindness and the FC of AI subregions within the searching voxels that showed statistically significant post hoc effects (q < 0.01, FDR correction)^11^.

## Additional Information

**How to cite this article**: Liu, L. *et al*. Visual deprivation selectively reshapes the intrinsic functional architecture of the anterior insula subregions. *Sci. Rep.* **7**, 45675; doi: 10.1038/srep45675 (2017).

**Publisher's note:** Springer Nature remains neutral with regard to jurisdictional claims in published maps and institutional affiliations.

## Supplementary Material

Supplementary Materials

## Figures and Tables

**Table 1:** Brain regions showing significant interaction between groups and AI locations.

Brain Region	Peak Z value	Cluster size	MNI coordinates (mm)		
			X	Y	Z
L.IPA	10.75	78	36	−63	57
R.IPA	9.68	63	−27	−69	54
L.CalS/L.LG	15.67	260	−21	−78	−18
R.CalS/R.LG	16.80	381	9	−75	−6
R.MOG	19.23	65	39	−87	18

Note: CalS = calcarine sulcus, IPA = intraparietal area, L = left, LG = lingual gyrus, MNI = Montreal Neurological Institute, MOG = middle occipital gyrus, R = right.
