# Supplementary material for: Visual deprivation selectively reshapes the intrinsic functional architecture of the anterior insula subregions
Source: Sci Rep. 2017 Mar 30;7:45675. doi: 10.1038/srep45675 (PMC5372462; doi:10.1038/srep45675)
Supplement: Supplementary Materials [file srep45675-s1.doc]

**Visual deprivation selectively reshapes the intrinsic functional architecture of the anterior insula subregions**

**Lihua Liu^1, 2^, Congcong Yuan^1^, Hao Ding^3^, Yongjie Xu^1^, Miaomiao Long^1, 2^, YanJun Li^1^, Yong Liu ^4,5,6,7^, Tianzi Jiang ^4,5,6,8,9^, Wen Qin^1^*, Wen shen^2^, Chunshui Yu^1^***

^1^ Department of Radiology, Tianjin Key Laboratory of Functional Imaging, Tianjin Medical University General Hospital, Tianjin 300052, China; ^2^Tianjin First Central Hospital, Tianjin 300192, China; ^3^School of Medical Imaging, Tianjin Medical University, Tianjin 300070, China; ^4^Brainnetome Center, ^5^National Laboratory of Pattern Recognition and, ^6^CAS Center for Excellence in Brain, Science and Intelligence Technology, Institute of Automation, Chinese Academy of Sciences, Beijing 100190, China, ^7^University of Chinese Academy of Sciences, Beijing, 100049, China，^8^Key Laboratory for NeuroInformation of the Ministry of Education, School of Life Science and Technology, University of Electronic Science and Technology of China, Chengdu 625014, China, ^9^The Queensland Brain Institute, University of Queensland, Brisbane, QLD 4072, Australia

***Correspondence to:** Chunshui Yu, or Wen Qin, Department of Radiology, Tianjin Medical University General Hospital, No. 154, Anshan Road, Heping District, Tianjin 300052, China. E-mail: [chunshuiyu@tijmu.edu.cn](mailto:chunshuiyu@tijmu.edu.cn) (C.Y.), [wayne.wenqin@gmail.com](mailto:wayne.wenqin@gmail.com) (W.Q.)

**Supplementary Tables**

Table S1: Demographic information of each blind subject

| Subjects | Gender | Age  (year) | Onset age  (year) | Handedness | Light perception | Braille begin age  (year) | Work | Work begin age  (year) | Mean FD | Spike % | Cause |
| --- | --- | --- | --- | --- | --- | --- | --- | --- | --- | --- | --- |
| CB001 | Male | 28 | 0 | right | weak | 17 | massagist | 17 | 0.13 | 0.00 | Retinal dysplasia |
| CB002 | Female | 28 | 0 | right | weak | 12 | massagist | 18 | 0.13 | 1.18 | Retinal pigmentosa |
| CB003 | Female | 27 | 0 | right | no | 12 | massagist | 16 | 0.10 | 0.00 | Optic atrophy |
| CB004 | Male | 23 | 0 | right | weak | None | massagist | 18 | 0.23 | 3.71 | Fundus illness |
| CB005 | Male | 24 | 0 | right | no | 17 | massagist | 17 | 0.33 | 2.47 | Eye dysplasia |
| CB006 | Female | 27 | 0 | right | weak | 10 | massagist | 15 | 0.14 | 0.00 | Retinal pigmentosa |
| CB007 | Female | 20 | 0 | right | no | 18 | massagist | 18 | 0.07 | 0.00 | Unknown |
| CB008 | Male | 22 | 0 | right | no | 12 | massagist | 17 | 0.15 | 1.29 | Retinal dystrophia |
| CB009 | Male | 30 | 0 | right | weak | 8 | community staff | 17 | 0.18 | 4.12 | Congenital cataract |
| CB010 | Male | 22 | 0 | right | weak | none | massagist | 18 | 0.25 | 4.65 | Congenital cataract |
| CB011 | Male | 27 | 0 | right | no | 11 | massagist | 23 | 0.12 | 2.35 | Optic atrophy |
| CB012 | Female | 20 | 0 | right | no | 17 | massagist | 17 | 0.08 | 0.00 | Microphthalmus |
| CB013 | Male | 23 | 0 | right | weak | 9 | massagist | 14 | 0.20 | 4.12 | Unknown |
| CB014 | Male | 39 | 0 | right | no | none | massagist | 22 | 0.31 | 4.71 | Pupil hypoplasia, microphthalmus |
| CB015 | Male | 36 | 0 | right | weak | none | massagist | 20 | 0.24 | 4.12 | Fundus hypoplasia |
| CB016 | Male | 29 | 0 | right | weak | 9 | massagist | 18 | 0.27 | 8.24 | Unknown |
| CB017 | Female | 21 | 0 | right | weak | 8 | student | never | 0.15 | 0.59 | Nystagmus, fundus hypoplasia |
| CB018 | Male | 31 | 0 | right | weak | 11 | massagist | 18 | 0.14 | 0.00 | Congenital microphthalmus |
| CB019 | Female | 27 | 0 | right | no | 21 | massagist | 21 | 0.15 | 0.00 | Congenital cataract |
| CB020 | Male | 28 | 0 | right | no | 9 | massagist | 17 | 0.15 | 0.00 | Fundus hypoplasia |
| EB001 | Male | 26 | 10 | right | weak | 14 | massagist | 18 | 0.20 | 4.12 | Congenital glaucoma |
| EB002 | Male | 25 | 10 | right | no | 10 | massagist | 19 | 0.10 | 0.00 | Congenital glaucoma |
| EB003 | Male | 37 | 2 | right | no | None | massagist | 27 | 0.28 | 5.29 | Unknown |
| EB004 | Male | 26 | 9 | right | no | None | massagist | 22 | 0.12 | 2.35 | Glaucoma |
| EB005 | Male | 27 | 10 | right | weak | None | massagist | 17 | 0.13 | 0.59 | Congenital microphthalmus |
| EB006 | Male | 31 | 9 | right | no | 19 | massagist | 20 | 0.14 | 1.18 | Retinal detached |
| EB007 | Male | 31 | 6 | right | weak | 9 | massagist | 14 | 0.18 | 0.00 | Congenital glaucoma |
| EB008 | Male | 20 | 6 | right | no | 11 | massagist | 16 | 0.18 | 1.18 | Fundus injury |
| EB009 | Male | 22 | 8 | right | weak | 12 | massagist | 16 | 0.08 | 0.00 | Optic atrophy |
| EB010 | Male | 23 | 9 | right | weak | None | massagist | 15 | 0.14 | 1.76 | Optic atrophy |
| EB011 | Male | 26 | 9 | right | no | 18 | massagist | 18 | 0.24 | 10.59 | Congenital microphthalumus |
| EB012 | Male | 22 | 12 | right | weak | 17 | massagist | 17 | 0.16 | 1.18 | Unknown |
| EB013 | Female | 24 | 2 | right | weak | 19 | massagist | 19 | 0.20 | 2.94 | Unknown |
| EB014 | Male | 23 | 11 | right | no | 9 | pianist | 18 | 0.14 | 0.00 | Glaucoma, cataract |
| EB015 | Female | 23 | 12 | right | weak | none | massagist | 22 | 0.14 | 0.00 | Cataract |
| EB016 | Female | 28 | 9 | right | no | 11 | community staff | 18 | 0.18 | 1.18 | Unknown |
| EB017 | Male | 41 | 8 | right | no | 18 | massagist | 18 | 0.20 | 0.00 | Unknown |
| EB018 | Male | 44 | 9 | right | no | 7 | massagist | 31 | 0.14 | 3.53 | Cataract |
| EB019 | Female | 45 | 10 | right | no | 8 | massagist | 14 | 0.12 | 0.00 | Unknown |
| EB020 | Male | 25 | 5 | right | no | 10 | massagist | 15 | 0.22 | 4.71 | Injury |
| EB021 | Male | 30 | 5 | right | no | 7 | massagist | 18 | 0.10 | 0.00 | Optic atrophy, retinal detached |
| EB022 | Male | 27 | 10 | right | no | 12 | massagist | 21 | 0.17 | 0.00 | Unknown |
| EB023 | Male | 26 | 10 | right | weak | 27 | massagist | 27 | 0.12 | 0.00 | Glaucoma |
| EB024 | Male | 23 | 12 | right | weak | 18 | massagist | 18 | 0.14 | 2.94 | Fundus hemorrhage |
| EB025 | Female | 24 | 8 | right | no | 21 | massagist | 21 | 0.13 | 0.00 | Retinopathy |
| EB026 | Female | 45 | 2 | right | weak | 12 | massagist | 30 | 0.21 | 0.00 | Leukoma, nystagmus |
| EB027 | Female | 35 | 3 | right | no | 27 | massagist | 27 | 0.19 | 0.59 | Unknown |
| LB001 | Male | 35 | 20 | right | no | 21 | massagist | 23 | 0.16 | 2.94 | Retinal detached |
| LB002 | Male | 26 | 22 | right | no | 17 | massagist | 19 | 0.17 | 0.00 | Congenital cataract |
| LB003 | Male | 39 | 22 | right | weak | 12 | massagist | 21 | 0.15 | 1.18 | Congenital microphthalmus |
| LB004 | Male | 24 | 18 | right | weak | 18 | massagist | 18 | 0.08 | 0.00 | Congenital amblyopia |
| LB005 | Male | 28 | 13 | right | weak | 15 | massagist | 18 | 0.13 | 0.00 | Retinal detached |
| LB006 | Male | 27 | 17 | right | no | 17 | massagist | 25 | 0.32 | 12.35 | Developmental glaucoma |
| LB007 | Male | 35 | 15 | right | no | None | massagist | 23 | 0.11 | 0.00 | Retinal detached |
| LB008 | Male | 39 | 32 | right | weak | 30 | massagist | 31 | 0.23 | 1.76 | Retinal pigmentosa |
| LB009 | Male | 27 | 14 | right | weak | 10 | massagist | 16 | 0.13 | 2.35 | Congenital glaucoma |
| LB010 | Male | 28 | 16 | right | no | 11 | massagist | 15 | 0.12 | 0.00 | Glaucoma, cataract |
| LB011 | Male | 25 | 21 | right | no | 21 | massagist | 21 | 0.15 | 4.71 | Optic dystrophia |
| LB012 | Male | 30 | 17 | right | no | 18 | massagist | 18 | 0.09 | 0.59 | Retinal detached |
| LB013 | Male | 43 | 20 | right | weak | None | massagist | 24 | 0.15 | 0.59 | Retinal detached |
| LB014 | Female | 25 | 17 | right | weak | 17 | massagist | 17 | 0.16 | 0.00 | Congenital microphthalmus, cataract |
| LB015 | Male | 29 | 25 | right | no | 25 | massagist | 25 | 0.15 | 0.00 | Ocular hypertension |
| LB016 | Female | 24 | 13 | right | no | 14 | massagist | 17 | 0.12 | 0.00 | Fundus haemorrhage |
| LB017 | Female | 28 | 16 | right | weak | 18 | massagist | 22 | 0.12 | 1.18 | Unknown, iridocyclitis |
| LB018 | Female | 30 | 20 | right | weak | 9 | massagist | 18 | 0.25 | 4.12 | Retinal pigmentosa |
| LB019 | Male | 26 | 15 | right | weak | none | massagist | 16 | 0.07 | 0.00 | Congenital cataract |
| LB020 | Male | 29 | 13 | right | no | 21 | massagist | 21 | 0.16 | 1.18 | Glaucoma |
| LB021 | Male | 33 | 28 | right | weak | 26 | massagist | 26 | 0.13 | 0.59 | Retinal pigmentosa |
| LB022 | Male | 27 | 14 | right | no | 18 | massagist | 18 | 0.21 | 5.29 | Retinal detached |
| LB023 | Female | 21 | 17 | right | weak | 19 | massagist | 20 | 0.25 | 4.12 | Fundus inflammation |
| LB024 | Male | 23 | 18 | right | weak | 19 | massagist | 19 | 0.11 | 0.00 | Optic atrophy |
| LB025 | Male | 41 | 15 | right | no | none | massagist | 32 | 0.18 | 2.94 | Glaucoma |
| LB026 | Male | 41 | 34 | right | weak | none | massagist | 17 | 0.11 | 0.00 | Optic atrophy,small corneal |
| LB027 | Female | 28 | 20 | right | weak | 25 | massagist | 23 | 0.16 | 1.76 | Congenital cataract |
| LB028 | Male | 30 | 17 | right | no | 27 | massagist | 27 | 0.19 | 7.65 | Retinal detached |
| LB029 | Female | 23 | 16 | right | weak | 20 | massagist | 20 | 0.19 | 1.18 | Congenital cataract |
| LB030 | Male | 31 | 24 | right | weak | 22 | massagist | 22 | 0.10 | 0.00 | Congenital cataract |
| LB031 | Female | 31 | 17 | right | no | 19 | massagist | 23 | 0.09 | 0.00 | Optic atrophy |
| LB032 | Female | 27 | 15 | right | weak | 19 | massagist | 19 | 0.16 | 1.76 | Unknown |
| LB033 | Female | 20 | 13 | right | weak | 15 | massagist | 14 | 0.11 | 0.00 | Unknown |
| LB034 | Female | 45 | 18 | right | no | 12 | massagist | 18 | 0.28 | 0.00 | Injury |
| LB035 | Male | 30 | 22 | right | weak | 13 | massagist | 19 | 0.09 | 0.00 | Retinopathy |
| LB036 | Female | 41 | 32 | right | weak | 39 | massagist | 32 | 0.11 | 0.00 | Retinal pigmentosa |
| LB037 | Male | 38 | 27 | right | weak | none | massagist | 27 | 0.17 | 0.00 | Optic atrophy |
| LB038 | Female | 34 | 25 | right | no | 25 | massagist | 26 | 0.19 | 0.00 | Fundus hemorrhage |
| LB039 | Female | 25 | 20 | right | weak | none | massagist | 20 | 0.23 | 0.00 | Glaucoma |
| LB040 | Male | 34 | 17 | right | no | none | massagist | 20 | 0.26 | 10.59 | Glaucoma |
| LB041 | Male | 31 | 18 | right | weak | 18 | massagist | 18 | 0.06 | 0.00 | Optic neuritis |

Note：Weak light perception means that the blind individual can perceive the strong light, but cannot judge any pattern vision of the object, such as the shape, location, and color, etc. Abbreviations: CB = congenitally blind, EB = early blind, FD = frame-wise displacement, LB = late blind.

**Supplementary Figures**


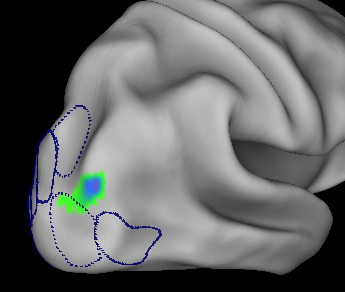


**Figure S1.** The adjacent relationships between the retinotopic areas and the MOG that showed decreased FC with the dorsal AI in the CB. The retinotopic areas are provided from PALS-12 altas.
